# Assessment of Hospital Characteristics and Interhospital Transfer Patterns of Adults With Emergency General Surgery Conditions

**DOI:** 10.1001/jamanetworkopen.2021.23389

**Published:** 2021-09-01

**Authors:** Cindy Y. Teng, Billie S. Davis, Matthew R. Rosengart, Kathleen M. Carley, Jeremy M. Kahn

**Affiliations:** 1 Department of Surgery, University of Pittsburgh Medical Center, Pittsburgh, Pennsylvania; 2 Department of Critical Care Medicine, University of Pittsburgh Medical Center, Pittsburgh, Pennsylvania; 3 Department of Computer Science, Carnegie Mellon University, Pittsburgh, Pennsylvania; 4 Department of Engineering, Carnegie Mellon University, Pittsburgh, Pennsylvania; 5 Department of Public Policy, Carnegie Mellon University, Pittsburgh, Pennsylvania; 6 Department of Health Policy and Management, University of Pittsburgh Graduate School of Public Health, Pittsburgh, Pennsylvania

## Abstract

**Importance:**

Although patients with emergency general surgery (EGS) conditions frequently undergo interhospital transfers, the transfer patterns and associated factors are not well understood.

**Objective:**

To examine whether patients with EGS conditions are consistently directed to hospitals with more resources and better outcomes.

**Design, Setting, and Participants:**

This cohort study performed a network analysis of interhospital transfers among adults with EGS conditions from January 1 to December 31, 2016. The analysis used all-payer claims data from the 2016 Healthcare Cost and Utilization Project state inpatient and emergency department databases in 8 states. A total of 728 hospitals involving 85 415 transfers of 80 307 patients were included. Patients were eligible for inclusion if they were 18 years or older and had an acute care hospital encounter with a diagnosis of an EGS condition as defined by the American Association for the Surgery of Trauma. Data were analyzed from January 1, 2020, to June 17, 2021.

**Exposures:**

Hospital-level measures of size (total bed capacity), resources (intensive care unit [ICU] bed capacity, teaching status, trauma center designation, and presence of trauma and/or surgical critical care fellowships), EGS volume (annual EGS encounters), and EGS outcomes (risk-adjusted failure to rescue and in-hospital mortality).

**Main Outcomes and Measures:**

The main outcome was hospital-level centrality ratio, defined as the normalized number of incoming transfers divided by the number of outgoing transfers. A higher centrality ratio indicated more incoming transfers per outgoing transfer. Multivariable regression analysis was used to test the hypothesis that a higher hospital centrality ratio would be associated with more resources, higher volume, and better outcomes.

**Results:**

Among 80 307 total patients, the median age was 63 years (interquartile range [IQR], 50-75 years); 52.1% of patients were male and 78.8% were White. The median number of outgoing and incoming transfers per hospital were 106 (IQR, 61-157) and 36 (IQR, 8-137), respectively. A higher log-transformed centrality ratio was associated with more resources, such as higher ICU capacity (eg, >25 beds vs 0-10 beds: β = 1.67 [95% CI, 1.16-2.17]; *P* < .001), and higher EGS volume (eg, quartile 4 [highest] vs quartile 1 [lowest]: β = 0.78 [95% CI, 0-1.57]; *P* = .01). However, a higher log-transformed centrality ratio was not associated with better outcomes, such as lower in-hospital mortality (eg, quartile 4 [highest] vs quartile 1 [lowest]: β = 0.30 [95% CI, −0.09 to 0.68]; *P* = .83) and lower failure to rescue (eg, quartile 4 [highest] vs quartile 1 [lowest]: β = −0.50 [95% CI, −1.13 to 0.12]; *P* = .27).

**Conclusions and Relevance:**

In this study, EGS transfers were directed to high-volume hospitals with more resources but were not necessarily directed to hospitals with better clinical outcomes. Optimizing transfer destination in the interhospital transfer network has the potential to improve EGS outcomes.

## Key Points

**Question:**

Are interhospital transfers of patients with emergency general surgery (EGS) conditions consistently directed to hospitals with more resources and better outcomes?

**Findings:**

In this cohort study using network analysis of interhospital transfers of 80 307 adults with EGS conditions, transfers were consistently directed to high-volume hospitals with more resources. However, transfers were not necessarily directed to hospitals with better outcomes (ie, lower risk-adjusted failure to rescue and lower in-hospital mortality).

**Meaning:**

The results of this study suggest that opportunities exist to improve the selection of transfer destinations for patients with EGS conditions, with potential to improve clinical outcomes for transferred patients.

## Teaser

This cohort study uses network analysis to examine whether interhospital transfers of patients with emergency general surgery conditions are consistently directed to hospitals with more resources and better clinical outcomes.

## Introduction

More than 3 million admissions of patients with emergency general surgery (EGS) conditions occur annually in the US, accounting for 7.1% of all hospitalizations nationally.^1,2^ Emergency general surgury conditions are also associated with high mortality and costs, placing substantial burden on the health care system.^1,3,4,5,6,7,8,9,10,11^ Interhospital transfers of patients with EGS conditions are common, occurring in up to 13% of all EGS encounters, but little is known about the transfer patterns within the network.^1,12^ Acute care hospitals with EGS services are not evenly distributed with regard to population density or need for care and, at present, there are no standardized guidelines in place to direct patients to those hospitals.^1,11,12^ This problem creates gaps in access to EGS care that have disproportionate consequences for underserved and rural communities, furthering a need for standardized interhospital transfer guidelines and quality measures.^6,7,12^ Benefits of organized regionalization of care that includes standardized triage guidelines and verified specialty-specific centers of excellence have been found for trauma and complex oncologic surgeries.^13,14,15^ To design successful EGS care delivery, a better understanding of current transfer patterns and associated hospital characteristics is needed to identify targets for quality improvement, including tools to aid transfer decision-making and the selection of destination hospitals.^12,16^

One way to examine existing EGS transfer patterns and associated hospital factors is through network analysis. Network analysis has been used to model the spread of infection in hospital social networks and to examine interhospital transfers of patients with myocardial infarction or critical care needs.^17,18,19,20,21,22,23,24^ In a network analysis framework, the extent to which patients are consistently transferred to high-resourced, high-volume, and high-performing hospitals would be both a measure of the network’s current performance and a strategy for identifying inefficiencies to improve outcomes through regional care delivery.

Therefore, we assessed interhospital transfer patterns for EGS in the US using a network analysis approach. We specifically examined the association between hospital characteristics (eg, size, resources, EGS volume, and outcomes) and transfer patterns within the network. We hypothesized that in a successful regionalized network, interhospital transfers of patients with EGS conditions would be directed to hospitals with high resources, volume, and performance.

## Methods

### Data Source

This study was approved by the University of Pittsburgh Institutional Review Board and deemed exempt from human subjects review under exemption 4 of US Code of Federal Regulations because the study used data that were publicly available or recorded in a manner in which participants could not be identified. The study followed the Strengthening the Reporting of Observational Studies in Epidemiology (STROBE) reporting guideline for cohort studies.^25^

We performed a retrospective cohort study using all-payer claims data from the 2016 Healthcare Cost and Utilization Project (HCUP) state inpatient and emergency department (ED) databases in 8 states (Arkansas, Florida, Maryland, Massachusetts, Nebraska, New York, Vermont, and Wisconsin). These states were chosen because they provide HCUP with unique patient identifiers that enabled us to track patients across hospitals over time. All inpatient hospital stays with discharge dates between January 1 and December 31, 2016, including inpatient admissions from the ED, are captured in the state inpatient databases^26^ and were defined as inpatient encounters. All ED visits between January 1 and December 31, 2016, that did not result in an admission at the same hospital are captured in the state ED databases^26^ and were defined as ED-only encounters. Both databases include patient demographic characteristics, admission and discharge times, diagnostic and procedural codes from the *International Classification of Diseases, Tenth Revision, Clinical Modification* (*ICD-10-CM*), patient outcomes, and hospital identifiers.^27^

### Identification of EGS Episodes of Care

We defined EGS episodes of care as temporally adjacent ED-only encounters and inpatient encounters that occurred in different hospitals, in which the inpatient encounter included at least 1 diagnosis of an EGS condition as defined by the American Association for the Surgery of Trauma.^3^ Based on published methods, patient encounters were considered temporally adjacent if the discharge date of the first hospital encounter was on the same day or the previous day of the admission date of the second hospital encounter.^17^ We used general equivalence mappings from the Centers for Medicare & Medicaid Services to convert previously defined EGS diagnostic codes^3^ from the *International Classification of Diseases, Ninth Revision, Clinical Modification* (*ICD-9-CM*) to the *ICD-10-CM* because the 2016 HCUP data included *ICD-10-CM* codes only.^27^

We included EGS episodes for patients 18 years and older that involved interhospital transfers from the ED (ie, ED to inpatient transfers) or the inpatient setting (ie, inpatient to inpatient transfers). The ED to inpatient transfers included ED to ED transfers that resulted in inpatient admission at the destination hospital. We identified operations and procedures based on *ICD-9-CM* procedural codes defined by the HCUP Surgery Flags software.^28^ Because EGS care includes both nonoperative and operative management as well as care received at both pretransfer and posttransfer facilities, patients who were operatively and nonoperatively managed were both included.

### Network Analysis of Hospital Centrality Measures

**Figure 1.**
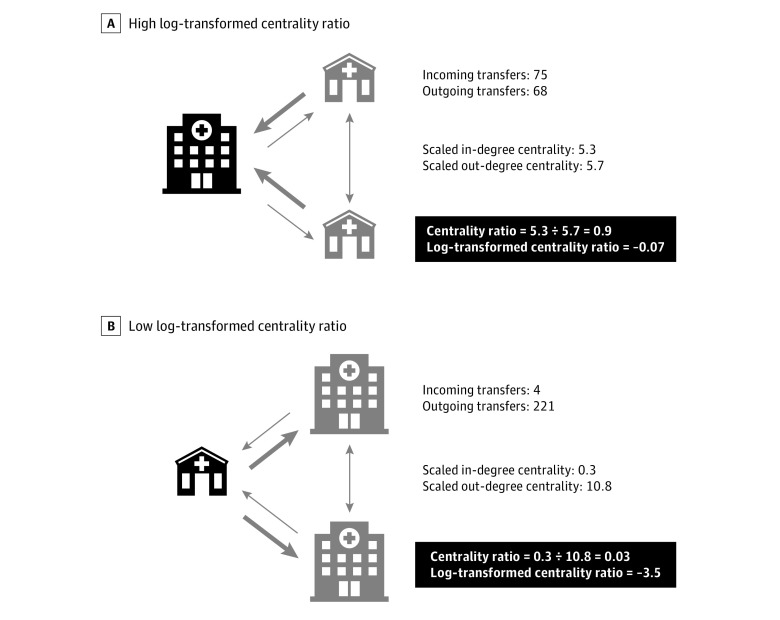
Examples of Hospitals With High and Low Log-Transformed Centrality Ratios

A network consists of nodes and directional links between nodes.^17,21,24,29,30,31^ In our networks, hospitals were considered nodes, and interhospital transfers were considered directional links between nodes, weighted by the number of transfers between the hospitals.^17,29,30,31^ We constructed 5 separate networks: (1) all transfers, (2) ED to inpatient transfers only, (3) inpatient to inpatient transfers only, (4) all transfers involving at least 1 operation or procedure, and (5) all transfers not involving any operation or procedure. For each network, we defined 3 centrality variables for each hospital: in-degree centrality (the normalized number of links directed to a node), out-degree centrality (the normalized number of links directed from a node), and centrality ratio (in-degree centrality divided by out-degree centrality, in which higher values indicated more incoming transfers per outgoing transfer)^29,30,31,32^ (Figure 1).

Centrality measures were normalized by the maximum possible number of links based on the number of nodes in the network. In-degree and out-degree centrality measures ranged from 0 to 1 and were scaled by multiplying by 10 000. Centrality measures were calculated based on standard methods used for network analysis.^30,31,32^ Based on standard definitions, in-degree centrality was calculated as the summation of links directed to a node in a given network, normalized by the maximum number of such links. Out-degree centrality was calculated as the summation of links directed away from a node in a given network, normalized by the maximum number of such links. Centrality ratio was calculated as in-degree centrality divided by out-degree centrality.

### Other Hospital Characteristics

#### Size and Resources

We obtained measures of hospital size and resources using the American Hospital Association Annual Survey,^33^ and we obtained trauma center designations from the trauma center listing of the American College of Surgeons.^34^ We identified the presence of trauma and/or surgical critical care fellowships using the fellowship listing from the Eastern Association for the Surgery of Trauma and the surgical critical care fellowship listing from the Accreditation Council for Graduate Medical Education as of December 20, 2019.^35,36^ We defined hospital size as the number of total hospital beds (categorized as 0-100 beds, 100-250 beds, and >250 beds). Measures of hospital resources included the number of intensive care unit (ICU) beds (categorized as 0-10 beds, 11-25 beds, and >25 beds), teaching status (categorized as nonteaching [defined as 0 full-time equivalent residents], small teaching [defined as full-time equivalent residents to total beds ratio of >0 and <0.2], and large teaching [defined as full-time equivalent residents to total beds ratio of ≥0.2]),^37,38^ trauma center designation (categorized as nontrauma, level 1, level 2, and level 3), and the presence of trauma and/or surgical critical care fellowships.

#### EGS Volume and Outcomes

We defined hospital EGS volume as the total number of inpatient EGS episodes that did not result in transfer within 1 calendar year. We defined hospital outcomes as risk-adjusted in-hospital mortality and failure to rescue for EGS episodes.^39,40^ Failure to rescue has been recognized by the National Quality Forum as a surgical quality measure.^14,40^ Using published methods, we calculated risk-adjusted in-hospital mortality and failure to rescue for each hospital while adjusting for patient age, sex, Elixhauser comorbidity index,^41^ and EGS diagnoses as defined by the American Association for the Surgery of Trauma.^3,39^ We did not adjust for whether a patient received an operation, as this factor is potentially endogenous to hospital quality.

Based on previous definitions, we defined failure to rescue as the occurrence of a complication and subsequent in-hospital death for EGS episodes that involved an operation or procedure; nonoperatively managed patients were excluded for this measure.^40,42^ We identified complications using *ICD-9-CM* diagnostic codes previously defined in the literature (eg, myocardial infarction, respiratory failure, pulmonary embolus or deep vein thrombosis, pneumonia, postoperative hemorrhage, surgical site infection, kidney failure, and shock).^40,42^ We identified operations and procedures (eg, laparotomy, laparoscopy, thoracotomy, endoscopic procedures, and percutaneous drainage) based on *ICD-9-CM* procedure codes defined by the HCUP Surgery Flags software.^28^ We again used the general equivalence mappings from the Centers for Medicare & Medicaid Services to convert diagnostic and procedural codes from the *ICD-9-CM* to the *ICD-10-CM*.^27^ These hospital outcome measures are specific to episodes among patients with EGS conditions. We categorized EGS volume, in-hospital mortality, and failure to rescue using quartiles, with quartile 1 indicating the lowest volume, mortality, or failure to rescue and quartile 4 indicating the highest.

### Statistical Analysis

We reported patient demographic characteristics and descriptive statistics of the overall EGS interhospital transfer networks. Using 1-way analysis of variance and post hoc pairwise comparisons, we compared hospital centrality ratio across the other hospital characteristics. For the primary analysis, we performed multivariable regression modeling with clustering by state, and we conducted partial *F* tests to examine the association of centrality ratio with measures of hospital resources, volumes, and outcomes (reported as β coefficients with 95% CIs). The centrality ratio was logarithmically transformed before conducting the analysis of variance and multivariable regression analysis because of nonnormal distribution. We performed sensitivity analyses using the same multivariable regression model for the following subnetworks: (1) ED to inpatient transfers, (2) inpatient to inpatient transfers, (3) transfers involving at least 1 operation or procedure, and (4) transfers not involving any operation or procedure.

Statistical significance was set at 2-tailed *P* < .05. We used ORA-PRO, version 3.0.9.9.100 (Netanomics), for network centrality calculation and Stata software, version 15 (StataCorp LLC), for statistical analyses. Data were analyzed from January 1, 2020, to June 17, 2021.

## Results

In 2016, 728 hospitals were involved in 85 415 transfers of 80 307 patients with EGS conditions in the 8 study states. The median age was 63 years (interquartile range [IQR], 50-75 years); 52.1% of patients were male, and 78.8% were White (Table 1^40,42^). Of the transfer episodes, 39.3% involved an operation or procedure at the referring hospital before transfer. The median number of outgoing and incoming transfers per hospital was 106 (IQR, 61-157) and 36 (IQR, 8-137), respectively (Table 1). The median risk-adjusted failure to rescue was 0.12 (IQR, 0.11-0.13). The median hospital centrality ratio (ie, the number of incoming transfers per outgoing transfer) was 0.21 (IQR, 0.03-0.93).

**Table 1.** Patient Demographic Characteristics and Hospital Characteristics of Interhospital Transfers in Emergency General Surgery

Characteristic	No. (%)
Patients	
Total patients, No.	80 307
Age, median (IQR), y	63 (50-75)
Sex	
Male	41 835 (52.1)
Female	38 472 (47.9)
Race/ethnicity^a^	
White	59 905 (78.8)
Black	10 627 (14.0)
Hispanic^b^	5569 (7.3)
Other^c^	5459 (7.2)
Insurance status^d^	
Government	57 518 (71.7)
Private	17 453 (21.8)
Self-pay	2672 (3.3)
Other	2619 (3.3)
Hospitals	
Total hospitals, No.	728
Total EGS encounters, median (IQR)	1017 (152-2395)
Outgoing transfers, median (IQR)	
Total	106 (61-157)
From the ED	55 (29-88)
From an inpatient hospitalization	44 (17-72)
Incoming transfers, median (IQR)^e^	
Total	36 (8-137)
From the ED	20 (4-72)
From an inpatient hospitalization	14 (3-57)
EGS-specific outcomes, median (IQR)^f^	
Risk-adjusted in-hospital mortality	0.05 (0.05-0.06)
Risk-adjusted failure to rescue^g^	0.12 (0.11-0.13)
Overall EGS interhospital transfer network, median (IQR)	
Scaled in-degree centrality	0.91 (0.08-4.02)
Scaled out-degree centrality	3.89 (2.09-5.87)
Centrality ratio^h^^,^^i^	0.21 (0.03-0.93)
ED to inpatient transfer network, median (IQR)	
Scaled in-degree centrality	0.66 (0.05-2.73)
Scaled out-degree centrality	2.60 (1.42-4.35)
Centrality ratio^h^^,^^j^	0.23 (0.02-1.09)
Inpatient to inpatient transfer network, median (IQR)	
Scaled in-degree centrality	0.56 (0.06-2.30)
Scaled out-degree centrality	2.44 (0.90-4.04)
Centrality ratio^h^^,^^k^	0.18 (0.04-0.72)

Abbreviations: ED, emergency department; EGS, emergency general surgery; IQR, interquartile range.

^a^ Data on race were available for 94.6% of patients.

^b^ Data on Hispanic ethnicity were available for 94.8% of patients.

^c^ Other races included Asian/Pacific Islander (830 patients), American Indian (271 patients), and other/not specified (4358 patients).

^d^ Insurance status data were available for 99.9% of patients.

^e^ Incoming transfer data were available for 628 hospitals.

^f^ EGS-specific outcome data were available for 726 hospitals.

^g^ Failure to rescue was defined as the occurrence of complication and subsequent in-hospital death for EGS episodes involving a procedure or operation. Complications were identified using codes from the *International Classification of Diseases, Ninth Revision, Clinical Modification* (*ICD-9-CM*) that were previously defined in the literature.^40,42^

^h^ Centrality ratio was defined as in-degree centrality divided by out-degree centrality. It is a hospital-level measure of the number of incoming transfers per outgoing transfers.

^i^ Based on 704 hospitals.

^j^ Based on 702 hospitals.

^k^ Based on 696 hospitals.

**Figure 2.**
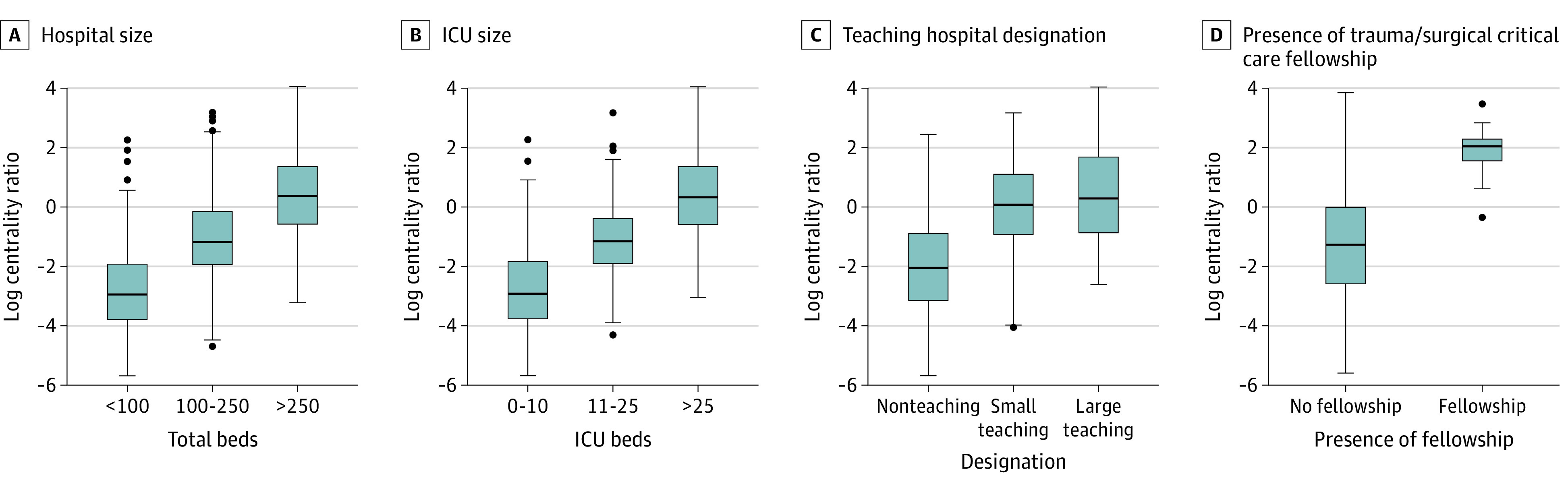
Hospital Log-Transformed Centrality Ratio for Emergency General Surgery Interhospital Transfers Based on Hospital Size, ICU Size, Teaching Hospital Status, and Presence of Fellowships A, *P* < .001 for both comparisons. B, *P* < .001 for both comparisons. C, *P* < .001 for comparison between nonteaching and small teaching hospital status. D, *P* = .007. In all panels, the lines within the gray boxes represent the medians, the gray boxes represent the interquartile ranges (IQRs), the whiskers represent all data within the 1.5 IQR from the nearest interquartile, and the dots represent outliers. ICU indicates intensive care unit.

**Figure 3.**
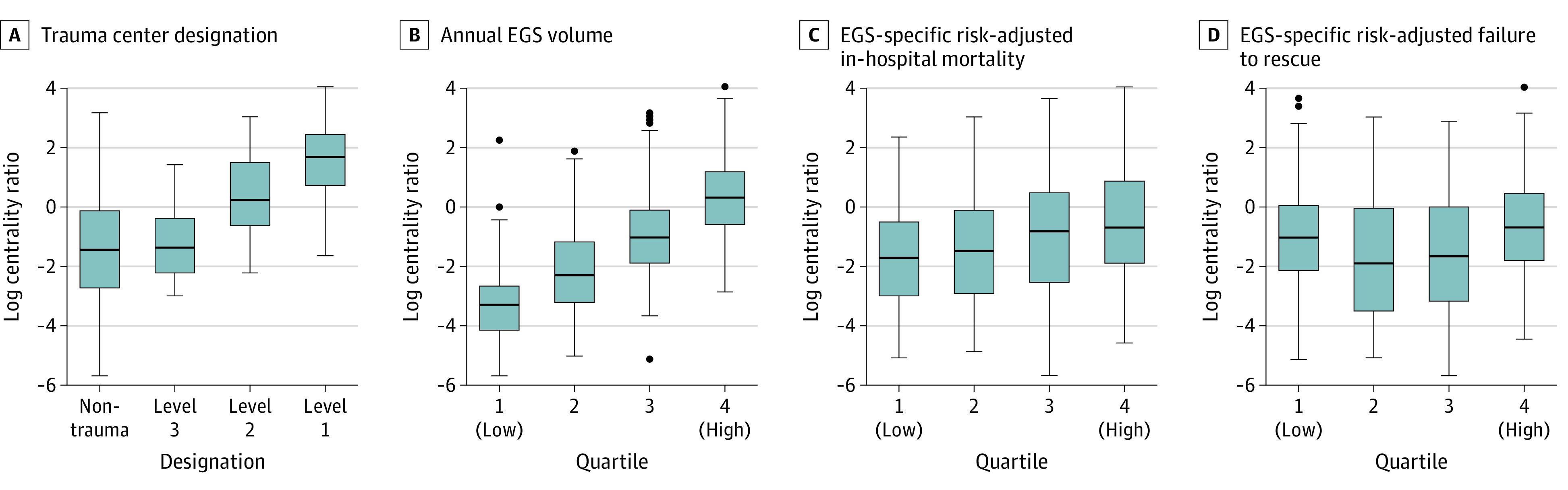
Hospital Log-Transformed Centrality Ratio for Emergency General Surgery Interhospital Transfers Based on Trauma Center Designation, Annual Volume, In-Hospital Mortality, and Failure to Rescue A, *P* = .007 for comparison between level 3 and level 2 trauma center designations. B, Quartile 1 indicates lowest volume, and quartile 4 indicates highest volume. *P* < .001 for all comparisons. C, Quartile 1 indicates lowest mortality, and quartile 4 indicates highest mortality. D, Quartile 1 indicates lowest failure to rescue, and quartile 4 indicates highest failure to rescue. *P* = .005 for comparison between quartile 1 and quartile 2; *P* < .001 for comparison between quartile 3 and quartile 4. In all panels, the lines within the gray boxes represent the medians, the gray boxes represent the interquartile ranges (IQRs), the whiskers represent all data within the 1.5 IQR from the nearest interquartile, and the dots represent outliers. EGS indicates emergency general surgery.

In the overall EGS interhospital transfer network, hospitals that were larger, with more resources and higher volume, generally had higher log-transformed centrality ratios (eg, >250 total beds vs <100 total beds: difference, 1.38 [95% CI, 1.05-1.71]; *P* < .001; >25 ICU beds vs 0-10 ICU beds: difference, 3.10 [95% CI, 2.80-3.40; *P* < .001; highest [quartile 4] vs lowest [quartile 1] EGS volume: difference, 3.61 [95% CI, 3.11-4.10]; *P* < .001), which indicated that they received more incoming transfers per outgoing transfer (Figure 2 and Figure 3). However, hospitals in the better outcome quartiles (eg, quartile 1 categories, indicating lowest failure to rescue and lowest in-hospital mortality) did not necessarily receive more incoming transfers per outgoing transfer (eg, lowest [quartile 1] vs highest [quartile 4] failure to rescue: difference, 0.37 [95% CI, −0.15 to 0.88]; *P* = .25; lower [quartile 1] vs higher [quartile 2] in-hospital mortality: difference, 0.33 [95% CI, −0.23 to 0.89]; *P* = .44). The log-transformed centrality ratio was significantly different across levels of hospital characteristics; the centrality ratio was higher for hospitals that were larger (eg, >250 total beds vs <100 total beds: difference, 1.38 [95% CI, 1.05-1.71]; *P* < .001) and had more resources (eg, >25 ICU beds vs 0-10 ICU beds: difference, 3.10 [95% CI, 2.80-3.40]; *P* < .001; large teaching vs nonteaching status: difference, 2.43 [95% CI, 2.02-2.84]; *P* < .001; level 1 trauma vs nontrauma designation: difference, 2.91 [95% CI, 2.16-3.66]; *P* < .001), and higher EGS volume (eg, highest [quartile 4] vs lowest [quartile 1]: difference, 3.61 [95% CI, 3.11-4.10]; *P* < .001) (Figure 2 and Figure 3). In the post hoc pairwise comparison, hospitals in successively higher in-hospital mortality quartiles (ie, quartile 1 vs quartile 2, quartile 2 vs quartile 3, and quartile 3 vs quartile 4) did not have a significantly higher log-transformed centrality ratio (eg, quartile 1 vs quartile 2: difference, 0.33 [95% CI, −0.23 to 0.89]; *P* = .44; quartile 2 vs quartile 3: difference, 0.42 [95% CI, −0.16 to 1.01]; *P* = .24; quartile 3 vs quartile 4: difference, 0.38 [95% CI, −0.16 to 0.92]; *P* = .26). Hospitals with the best EGS outcomes did not have significantly more incoming transfers per outgoing transfer compared with hospitals with the worst EGS outcomes. For example, hospitals with the lowest failure to rescue (quartile 1) vs the highest failure to rescue (quartile 4) had a mean (SD) log centrality ratio of −0.98 (1.72) vs −0.61 (1.68), representing a difference in log centrality ratio of 0.37 (95% CI, –0.15 to 0.88; *P* = .25).

In the primary multivariable analyses, a higher hospital log-transformed centrality ratio was associated with more hospital resources (eg, >25 ICU beds vs 0-10 ICU beds: β = 1.67 [95% CI, 1.16-2.17]; *P* < .001; large teaching vs nonteaching status: β = 0.68 [95% CI, 0.38-0.99]; *P* < .001) and higher EGS volume (eg, highest [quartile 4] vs lowest [quartile 1]: β = 0.78 [95% CI, 0-1.57]; *P* = .01) (Table 2). However, a higher log-transformed centrality ratio was not associated with measures of better outcomes (eg, highest [quartile 4] vs lowest [quartile 1] failure to rescue: β = −0.50 [95% CI, −1.13 to 0.12]; *P* = .27; highest [quartile 4] vs lowest [quartile 1] in-hospital mortality: β = 0.30 [95% CI, −0.09 to 0.68]; *P* = .83) (Table 2).

**Table 2.** Adjusted Association Between Hospital Characteristics and Log-Transformed Centrality Ratio for All Emergency General Surgery Interhospital Transfers

Characteristic	β coefficient (95% CI)	*P* value
Total beds		.34
0-100	1 [Reference]	
100-250	0.40 (–0.21 to 1.01)	
>250	0.60 (–0.31 to 1.51)	
ICU beds		<.001
0-10	1 [Reference]	
11-25	0.99 (0.65 to 1.33)	
>25	1.67 (1.16 to 2.17)	
Trauma center level		.25
Nontrauma	1 [Reference]	
3	–0.46 (–0.99 to 0.06)	
2	0.46 (–1.06 to 1.98)	
1	0.87 (–0.32 to 2.06)	
Trauma and/or surgical critical care fellowships		.19
No fellowships	1 [Reference]	
Fellowships	0.75 (–0.46 to 1.97)	
Teaching hospital status		<.001
Nonteaching	1 [Reference]	
Small teaching	0.70 (0.25 to 1.15)	
Large teaching	0.68 (0.38 to 0.99)	
EGS volume		.01
Quartile		
1 (Lowest volume)	1 [Reference]	
2	0.68 (0.37 to 0.99)	
3	0.61 (0.18 to 1.03)	
4 (Highest volume)	0.78 (0 to 1.57)	
Risk-adjusted in-hospital mortality		.83
Quartile		
1 (Lowest mortality)	1 [Reference]	
2	0.25 (–0.05 to 0.56)	
3	0.28 (–0.04 to 0.61)	
4 (Highest mortality)	0.30 (–0.09 to 0.68)	
Risk-adjusted failure to rescue		.27
Quartile		
1 (Lowest failure)	1 [Reference]	
2	–0.19 (–0.72 to 0.35)	
3	–0.09 (–0.66 to 0.48)	
4 (Highest failure)	–0.50 (–1.13 to 0.12)	

Abbreviations: EGS, emergency general surgery; ICU, intensive care unit.

The sensitivity analyses yielded mostly similar results for the EGS interhospital transfer networks, regardless of transfer origin (ED vs inpatient) or the involvement of an operation or procedure. For all 4 subgroups (ED to inpatient, inpatient to inpatient, with operation or procedure, and without operation or procedure), a higher log-transformed centrality ratio was associated with greater resources, including more ICU beds and teaching hospital status (eTable 1 and eTable 2 in the Supplement). Of note, higher EGS volume was not associated with a higher log-transformed centrality ratio for ED to inpatient transfers, but the volume-centrality ratio association was maintained for inpatient to inpatient transfers (eTable 1 in the Supplement). In addition, higher EGS volume was not associated with a higher log-transformed centrality ratio for transfers involving operations or procedures, but the volume-centrality ratio association was maintained for transfers not involving an operation or procedure (eTable 2 in the Supplement). Nevertheless, for all subgroups, a higher log-transformed centrality ratio was again not associated with better outcomes (ie, lower failure to rescue and lower in-hospital mortality) (eTable 1 and eTable 2 in the Supplement).

## Discussion

This cohort study was based on the concept that understanding existing interhospital transfer patterns is an important first step to guide targeted improvement strategies for the design of successful regionalized care and to improve outcomes for EGS. Yet the interhospital transfer network for EGS is not well understood, including the extent to which patients are transferred to hospitals with high resources, volume, and performance, as expected in a well-coordinated regionalized network. Applying a network analysis approach, we found that patients with EGS conditions were generally transferred to hospitals with high resources and volume, as expected. However, patients with EGS conditions were not consistently transferred to hospitals with better outcomes, as measured by lower risk-adjusted EGS in-hospital mortality and failure to rescue. These findings highlight the need for further research into the appropriate selection of transfer destinations as a strategy to improve EGS outcomes.^16^

Network analysis has been used to examine overall interhospital transfers, ICU transfers, and transfers of patients with acute myocardial infarction,^17,18,19,20,21,31^ but the approach in the examination of interhospital transfers for EGS is, to our knowledge, novel. In a regional critical care transfer network, higher centrality was associated with more hospital resources.^17^ Our results revealed parallel associations between a higher centrality ratio and greater hospital resources and EGS volume in the EGS transfer network. However, hospitals that received more incoming transfers per outgoing transfer (ie, hospitals with a higher centrality ratio) were not always those with better risk-adjusted EGS outcomes.

Hospital characteristics, such as higher ICU capacity, higher volume, and higher-quality trauma care, are associated with better EGS outcomes.^43,44,45,46^ In this context, clinicians initiating transfers might make informed decisions and choose destination hospitals that have more resources or better outcomes, which would have consequences for that hospital’s centrality ratio. However, existing studies on transfer decision-making have focused on factors associated with a clinician’s initial decision to transfer a patient.^47,48,49,50^ The process through which a clinician selects the most appropriate transfer destination hospital and the underlying factors associated with the clinician’s decision-making are largely unknown and likely multifactorial.^16^ These factors could include clinical and socioeconomic conditions, financial considerations such as insurance status, established interinstitutional partnerships, and logistic limitations such as bed availability.^1,16,51,52,53,54,55^

In interviews with clinicians at community hospitals, rapid transfer acceptance was reported to be more important than the relevant qualifications of hospitals, suggesting that logistic considerations may be prioritized over clinical hospital characteristics in the selection of transfer destinations.^51^ In addition, transfer patterns were often associated with established interhospital relationships rather than conscious efforts to optimize care for individual patients,^18,52^ suggesting that preexisting institutional practices and heuristics may be more important than individualized care when making transfer decisions. These findings and our results regarding transfer patterns highlight the need for further research on factors underlying transfer destination decisions to improve how we direct patients to appropriate hospitals for optimal care, which is an often overlooked aspect of interhospital transfers. These factors are modifiable targets for intervention that could potentially be addressed through the development of validated guidelines and risk stratification tools to aid physician decision-making. Our results raise questions about whether EGS transfers are consistently directed to appropriate hospitals for further care and new concerns regarding how we determine appropriate hospitals in the absence of standardized guidelines.

### Limitations

This study has limitations. Administrative data sets lack information such as patient physiologic status, reason for transfer, and long-term outcomes. Although the reason for transfer and transfer decision-making are important areas of research, they were not within the scope of our study’s primary objective. Further research could use different methodological approaches and instruments, such as qualitative interviews and surveys, to examine transfer decisions. Nevertheless, our sample includes all-payer claims data from multiple hospitals and states across different geographical regions, with transfers from both ED and inpatient settings, to provide a broader perspective of EGS transfer patterns. These results are applicable to adult patients with EGS conditions as defined by the American Association for the Surgery of Trauma. Although we collected data from multiple sources to examine more comprehensive hospital characteristics, information such as bed availability at destination hospitals during the time of transfer and the availability of general surgeons or specialists required for comprehensive EGS care is lacking. Future studies to examine the associations of bed availability and EGS staffing with interhospital transfer patterns would be valuable.

Our measures of hospital outcomes might be subject to discharge bias, a phenomenon in which patients with complex medical conditions are transferred before death, which artificially lowers mortality rates.^56,57,58^ However, most EGS episodes of care (93.6%) used to calculate our hospital-level risk-adjusted outcomes were episodes that did not involve interhospital transfer, making discharge bias less applicable. In addition, because each hospital may have different established interinstitutional relationships and collaboration with local emergency transport services, further region-specific research on transfer guidelines, and multidisciplinary coordination among clinicians, hospitals, and call centers is needed to tailor interventions to their respective health care networks.

## Conclusions

Interhospital transfers in EGS were consistently directed to high-volume hospitals with more resources but not necessarily to hospitals with better risk-adjusted outcomes. Further research to develop guidelines and infrastructure to improve transfer decisions and the appropriate selection of transfer destinations on both the clinician and health care system levels is needed and could improve outcomes for patients with EGS conditions.

## Notes

Supplement. **eTable 1.** Adjusted Association Between Hospital Characteristics and Log-Transformed Centrality Ratio for ED to Inpatient and Inpatient to Inpatient EGS Interhospital Transfers **eTable 2.** Adjusted Association Between Hospital Characteristics and Log-Transformed Centrality Ratio for EGS Interhospital Transfers With and Without Operations or Procedures
