# Supplementary material for: Assessment of Hospital Characteristics and Interhospital Transfer Patterns of Adults With Emergency General Surgery Conditions
Source: JAMA Netw Open. 2021 Sep 1;4(9):e2123389. doi: 10.1001/jamanetworkopen.2021.23389 (PMC8411299; doi:10.1001/jamanetworkopen.2021.23389)
Supplement: Supplement. — eTable 1. Adjusted Association Between Hospital Characteristics and Log-Transformed Centrality Ratio for ED to Inpatient and Inpatient to Inpatient EGS Interhospital Transfers eTable 2. Adjusted Association Between Hospital Characteristics and Log-Transformed Centrality Ratio for EGS Interhospital Transfers With and Without Operations or Procedures [file jamanetwopen-e2123389-s001.pdf]

Supplementary Online Content

Teng CY, Davis BS, Rosengart MR, Carley KM, Kahn JM. Assessment of hospital characteristics and interhospital transfer patterns of adults with emergency general surgery conditions. *JAMA Netw Open*. 2021;4(9):e2123389. doi:10.1001/jamanetworkopen.2021.23389

eTable 1. Adjusted Association Between Hospital Characteristics and Log-Transformed Centrality Ratio for ED to Inpatient and Inpatient to Inpatient EGS Interhospital Transfers

eTable 2. Adjusted Association Between Hospital Characteristics and Log-Transformed Centrality Ratio for EGS Interhospital Transfers With and Without Operations or Procedures

This supplementary material has been provided by the authors to give readers additional information about their work.

© 2021 Teng CY et al. *JAMA Network Open*.

eTable 1. Adjusted Association Between Hospital Characteristics and Log-Transformed Centrality Ratio for ED to Inpatient and Inpatient to Inpatient EGS Interhospital Transfers

	ED-Inpatient		Inpatient-Inpatient	
**Hospital Characteristics**	**Coefficient [95% CI]**	**p-value**	**Coefficient [95% CI]**	**p-value**
Total hospital beds				
0-100 beds	Referent	0.54	Referent	0.14
100 to 250 beds	0.34 [-0.39, 1.07]		0.24 [-0.30, 0.78]	
>250 beds	0.51 [-0.54, 1.55]		0.59 [-0.22, 1.40]	
ICU beds				
0-10 beds	Referent	<0.001	Referent	<0.001
11-25 beds	0.97 [0.52, 1.42]		0.80 [0.50, 1.09]	
>25 beds	1.74 [1.19, 2.30]		1.50 [0.96, 2.03]	
Trauma center level				
Non-trauma center	Referent	0.18	Referent	0.22
Level 3	-0.47 [-0.92, -0.02]		-0.58 [-1.21, 0.05]	
Level 2	0.70 [-0.95, 2.35]		0.15 [-1.08, 1.38]	
Level 1	0.91 [-0.26, 2.08]		0.89 [-0.34, 2.12]	
Trauma/Surgical Critical Care fellowship				
No fellowship	Referent	0.12	Referent	0.33
Has fellowship	0.82 [-0.26, 1.89]		0.82 [-0.26, 1.89]	
Teaching status				
Non-teaching	Referent	0.003	Referent	<0.001
Small teaching	0.60 [0.25, 0.94]		0.57 [0.21, 0.92]	
Large teaching	0.54 [0.15, 0.92]		0.67 [0.28, 1.05]	
EGS volume				
Quartile 1, lowest EGS volume	Referent	0.15	Referent	0.005
Quartile 2	1.01 [0.12, 1.90]		-0.77 [-1.11, -0.44]	
Quartile 3	1.13 [0.05, 2.22]		-1.03 [-1.63, -0.44]	
Quartile 4, highest EGS volume	1.27 [-0.13, 2.67]		-0.83 [-1.72, 0.07]	
Risk-adjusted in-hospital mortality				
Quartile 1, lowest mortality	Referent	0.27	Referent	0.63
Quartile 2	0.31 [-0.27, 0.88]		0.19 [-0.49, 0.85]	
Quartile 3	0.45 [-0.20, 1.10]		0.21 [-0.32, 0.75]	
Quartile 4, highest mortality	0.37 [-0.38, 1.11]		0.26 [-0.38, 0.90]	
Risk-adjusted failure to rescue				
Quartile 1, lowest failure to rescue	Referent	0.12	Referent	0.06
Quartile 2	-0.20 [-0.67, 0.27]		-0.18 [-0.61, 0.25]	
Quartile 3	-0.11 [-0.80, 0.58]		0.05 [-0.46, 0.57]	
Quartile 4, highest failure to rescue	-0.53 [-1.11, 0.05]		-0.48 [-1.10, 0.15]	

Abbreviations: ED=emergency department; CI = confidence interval; ICU = intensive care unit; EGS = emergency general surgery.

© 2021 Teng CY et al. *JAMA Network Open*.

eTable 2. Adjusted Association Between Hospital Characteristics and Log-Transformed Centrality Ratio for EGS Interhospital Transfers With and Without Operations or Procedures

	With Operation/Procedure		Without Operation/Procedure	
**Hospital Characteristic**	**Coefficient [95% CI]**	**p-value**	**Coefficient [95% CI]**	**p-value**
Total hospital beds				
0-100 beds	Referent	0.43	Referent	0.32
100 to 250 beds	0.11 [-0.50, 0.71]		0.43 [-0.20, 1.06]	
>250 beds	0.38 [-0.58, 1.33]		0.57 [-0.37, 1.51]	
ICU beds				
0-10 beds	Referent	<0.001	Referent	<0.001
11-25 beds	1.06 [0.63, 1.49]		0.89 [0.49, 1.29]	
>25 beds	1.87 [1.23, 2.51]		1.51 [0.94, 2.08]	
Trauma center level				
Non-trauma center	Referent	0.22	Referent	0.33
Level 3	-0.69 [-1.45, 0.06]		-0.34 [-0.78, 0.10]	
Level 2	0.51 [-1.13, 2.15]		0.46 [-1.03, 1.94]	
Level 1	1.13 [-0.21, 2.47]		0.74 [-0.32, 1.80]	
Trauma/Surgical Critical Care fellowship				
No fellowship	Referent	0.22	Referent	0.18
Has fellowship	0.85 [-0.63, 2.34]		0.65 [-0.40, 1.70]	
Teaching status				
Non-teaching	Referent	<0.001	Referent	<0.001
Small teaching	0.68 [0.28, 1.07]		0.65 [0.21, 1.09]	
Large teaching	0.64 [0.17, 1.11]		0.62 [0.32, 0.92]	
EGS volume				
Quartile 1, lowest EGS volume	Referent	0.51	Referent	0.01
Quartile 2	-0.14 [-0.63, 0.34]		0.68 [0.30, 1.05]	
Quartile 3	-0.29 [-1.11, 0.53]		0.71 [0.27, 1.15]	
Quartile 4, highest EGS volume	0.07 [-0.91, 1.05]		0.80 [-0.03, 1.64]	
Risk-adjusted in-hospital mortality				
Quartile 1, lowest mortality	Referent	0.59	Referent	0.48
Quartile 2	0.30 [-0.42, 1.02]		0.34 [-0.23, 0.91]	
Quartile 3	0.34 [-0.41, 1.10]		0.35 [-0.36, 1.06]	
Quartile 4, highest mortality	0.48 [-0.36, 1.32]		0.35 [-0.42, 1.11]	
Risk-adjusted failure to rescue				
Quartile 1, lowest failure to rescue	Referent	0.18	Referent	0.22
Quartile 2	-0.12, [-0.64, 0.40]		-0.18 [-0.71, 0.35]	
Quartile 3	-0.18 [-0.82, 0.46]		-0.12 [-0.72, 0.48]	
Quartile 4, highest failure to rescue	-0.70 [-1.44, 0.05]		-0.50 [-1.16, 0.15]	

Abbreviations: CI = confidence interval; ICU = intensive care unit; EGS = emergency general surgery.

© 2021 Teng CY et al. *JAMA Network Open*.
